# Genome-Wide Identification of the Caffeoyl Coenzyme A 3-O-Methyltransferase Family Genes and Their Role in the Lodging Resistance of Wheat (*Triticum aestivum* L.)

**DOI:** 10.3390/plants15192996

**Published:** 2026-09-30

**Authors:** Chunhui Li, Shufang Sun, Jing Wang, Jiayu Wang, Haixing Cui

**Affiliations:** 1 State Key Laboratory of Wheat Improvement, College of Agronomy, Shandong Agricultural University, Tai’an 271000, China; chunhuili@sdau.edu.cn (C.L.); sun17653475426@163.com (S.S.); 17658156790@163.com (J.W.); wangjiayu0817@163.com (J.W.); 2 Cryo-Electron Microscopy Center, Shandong Agricultural University, Tai’an 271000, China

**Keywords:** wheat, genome-wide identification, *TaCCoAOMT* gene family, lignin, lodging resistance

## Abstract

As an enzyme, Caffeoyl coenzyme A 3-O-methyltransferase (CCoAOMT) plays a crucial role in lignin synthesis. In this study, we identified 21 *TaCCoAOMTs* in the wheat genome and, based on phylogenetic analysis, categorized them into four primary groups. *TaCCoAOMTs* within the same branch exhibited exon–intron structures and motif compositions similar to one another. Collinearity analysis showed that both segmental and tandem duplication contributed to the expansion of the *TaCCoAOMT* family in wheat. Several cis-elements associated with “growth and development” and “phytohormone response” were found in the promoter regions of *TaCCoAOMTs*. By analyzing *TaCCoAOMT* expression across tissues, we identified seven genes with significantly elevated expression in highly lignified tissues. We also compared *TaCCoAOMT* expression levels among four wheat varieties with varying degrees of lodging resistance. *TaCCoAOMT10* and *TaCCoAOMT18* exhibited significantly higher expression in lodging-resistant varieties than in susceptible ones and are therefore considered candidate genes associated with lodging resistance. Lodging-resistant varieties showed greater breaking strength and higher lignin content. These findings lay the groundwork for understanding TaCCoAOMT functions in wheat and for breeding programs aimed at improving lodging resistance.

## 1. Introduction

Wheat (*Triticum aestivum* L.) provides food for one-third of the global population, and because world food demand is forecast to rise by 60% by 2050, production of this crop will have to increase by more than 50% over today’s levels [1]. However, annual wheat yield loss due to lodging is astonishing, ranging from 11% to 75%, causing severe economic losses in wheat cultivation [2]. Lodging, triggered by various factors, shifts the plant body vertically [3]. Lodging can be classified into stem and root lodging [4]. Stem lodging is the most common, mainly occurring at the basal second internode [5]. Lodging not only reduces crop yield and quality but also makes harvesting more difficult [6]. Therefore, breeding lodging-resistant wheat varieties is an efficient method to improve wheat yield.

The stem receives its mechanical strength from the cell wall, which is primarily composed of cellulose, hemicellulose, and lignin [7]. Lignin, a major constituent of secondary cell walls, improves both the physical and mechanical properties of plants by increasing stem stiffness and breaking strength [8]. Lignin is a complex macromolecule derived from the polymerization of three monomer types: syringyl (S), guaiacyl (G), and hydroxyphenyl (H) units [9]. In herbaceous plants, the S and G monomers are the most abundant [10]. Previous studies show that S and G monomers positively affect stem lodging resistance [11,12]. Compared to lodging-sensitive wheat cultivars, lodging-resistant wheat cultivars have a more significant amount of S and G monomers [13]. Regulating critical genes involved in the synthesis of lignin S and G monomers can effectively enhance stem lodging resistance.

Lignin monomers are biosynthesized sequentially through the shikimic acid pathway, the phenylpropanoid pathway, and the specific lignin pathway. Phenylalanine undergoes a series of reactions, such as deamination, hydroxylation, methylation, and redox reactions, to produce three monomers [14,15]. Caffeoyl coenzyme A 3-O-methyltransferase (CCoAOMT) is a key methyltransferase that catalyzes the transfer of a methyl group from S-adenosyl-L-methionine to the C5 position of the benzene ring in caffeoyl-CoA and 5-hydroxyferuloyl-CoA, thereby contributing to the biosynthesis of S and G monomers [16]. This reaction yields feruloyl-CoA and sinapoyl-CoA, respectively [17,18]. In rice, mutations in the *OsCCoAOMT* gene reduce accumulation of S and G monomers, decreasing total lignin [19]. Multiple studies also show that mutations in the *CCoAOMT* gene affect the S/G ratio [20].

Yang et al. characterized the wheat *CCoAOMT* gene family and screened candidate genes associated with disease resistance [21], whereas the present study identifies family members contributing to wheat lodging resistance. In this study, we conducted a comprehensive analysis of the *TaCCoAOMT* genes. We identified 21 *TaCCoAOMT* members in wheat (*Triticum aestivum* L.) and evaluated their biophysical properties, gene structures, conserved motifs, chromosomal distribution, and collinearity analysis. To elucidate the relationship between *TaCCoAOMT* genes and lodging resistance, we analyzed their expression patterns. We further assessed the relationships between *TaCCoAOMT* expression and key lodging-related traits—including stem breaking strength, lignin deposition, and monolignol composition—using both lodging-tolerant and lodging-susceptible wheat cultivars. These results help us better understand the potential functions of *TaCCoAOMT* genes. They also identify candidate genes to improve wheat stem lodging resistance.

## 2. Results

### 2.1. Identification of CCoAOMT Genes in Wheat

Using HMM profiles of the SAM-dependent methyltransferase domain (PF01596), we screened the wheat genome and identified 21 *CCoAOMT* genes, which were designated *TaCCoAOMT1* through *TaCCoAOMT21*. A comparative genomic search further revealed six and seven homologous genes in rice and Arabidopsis, respectively (Table S1). These TaCCoAOMT proteins show distinct physicochemical properties, with predicted molecular weights ranging from 16.28 kDa to 34.16 kDa (Table S2). Meanwhile, TaCCoAOMT proteins have isoelectric points (pI) ranging from 4.94 to 11.11, as well as instability indices ranging from 25.39 to 85.66.

### 2.2. Phylogenetic Analysis of the CCoAOMTs in Different Species

**Figure 1:**
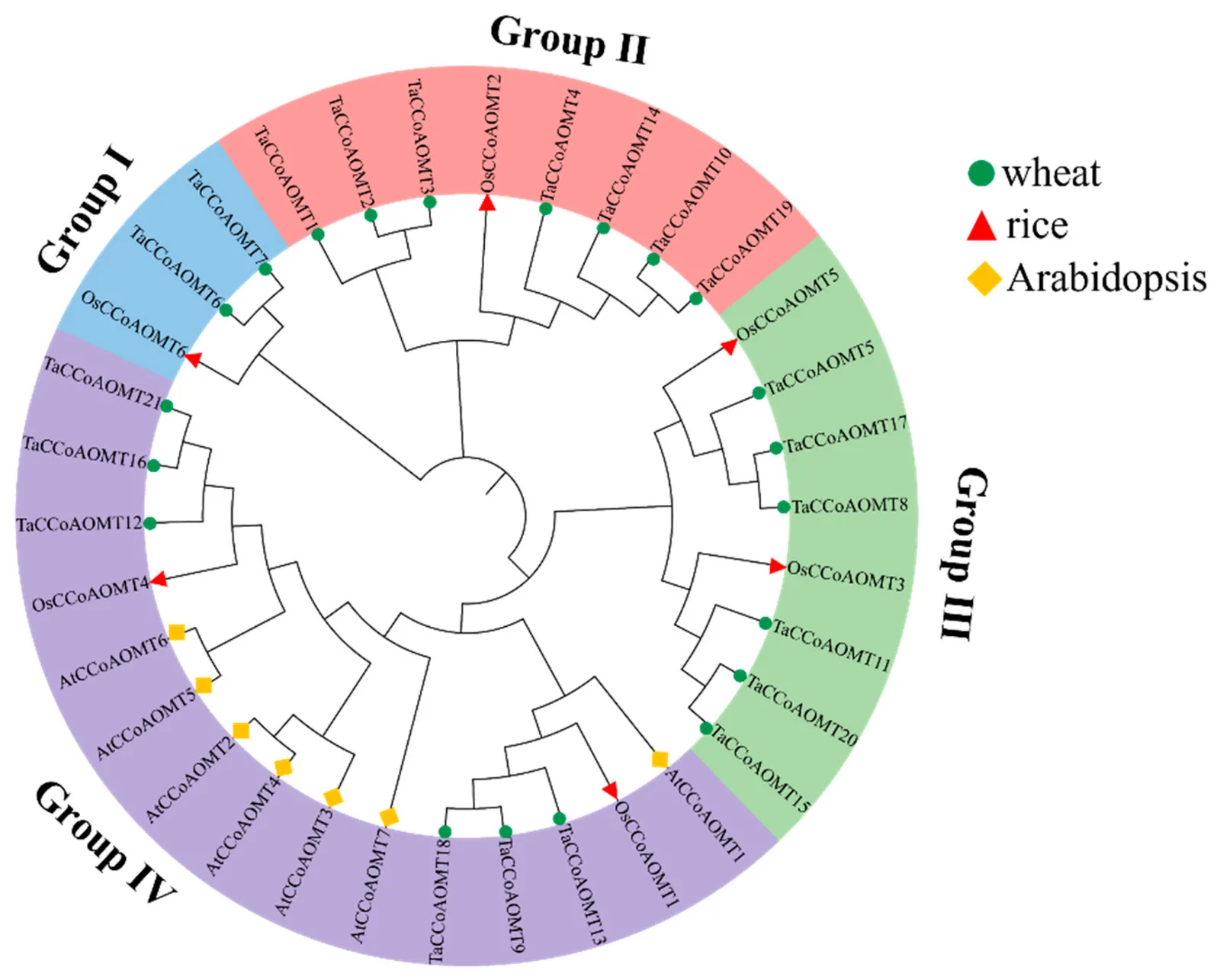
The phylogenetic relationship of CCoAOMT proteins in *Triticum aestivum*, *Oryza sativa*, and *Arabidopsis thaliana*. The evolutionary tree was constructed by MEGA11.

To analyze the evolutionary relationship of the *CCoAOMT* family in wheat, we constructed a phylogenetic tree of CCoAOMTs in wheat, rice, and Arabidopsis (Figure 1). The CCoAOMTs were clustered into four subfamilies based on their sequence alignment. Among them, Group IV was the largest subfamily, with 15 CCoAOMT proteins. Group I contained only three genes, and all AtCCoAOMTs clustered in Group IV. The evolutionary tree showed that multiple TaCCoAOMTs cluster closely with OsCCoAOMTs, indicating homology and similar gene functions.

### 2.3. Gene Structure and Conserved Motifs of TaCCoAOMT Genes

**Figure 2:**
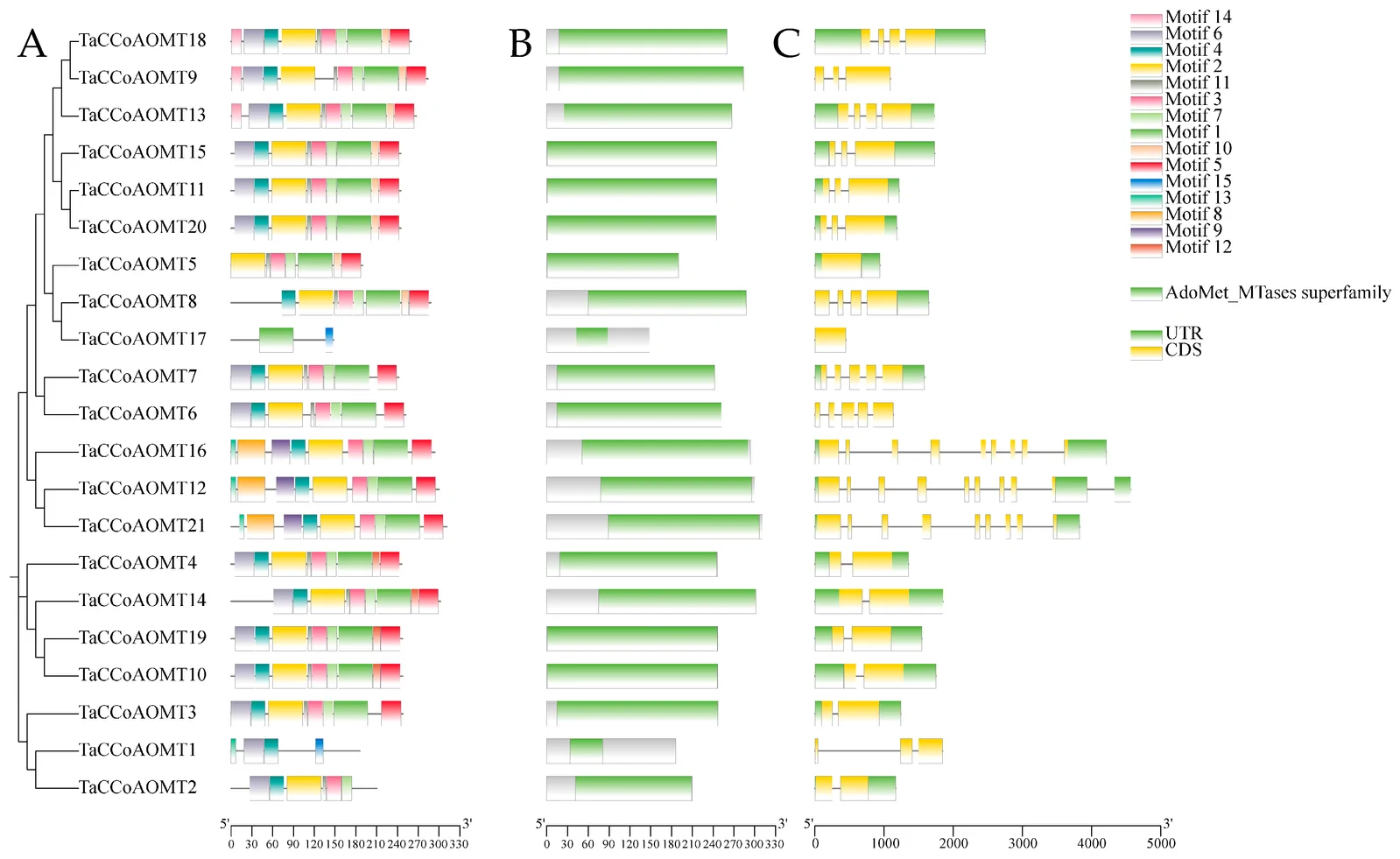
Conserved motifs, conserved domain, and gene structure of *TaCCoAOMTs* in wheat. (**A**) Motifs distribution of *TaCCoAOMT* genes. (**B**) Conserved domain analysis of *TaCCoAOMTs* in wheat. (**C**) Exon–intron structure of *TaCCoAOMT* genes. CDS, coding sequence for protein; UTR, untranslated region.

Gene structure information is valuable for inferring evolutionary relationships and assigning subfamily classifications [22]. The results showed that 15 motifs were identified in the *TaCCoAOMTs*. Most members of the *TaCCoAOMT* family typically contain 6 to 10 motifs, while *TaCCoAOMT17* and *TaCCoAOMT1* only had 2 and 4 motifs, respectively (Figure 2A). Motifs 1 and 2, consisting of 50 amino acids, have the most extended motifs, whereas motifs 11 and 13, respectively, consist of 6 and 8 amino acids (Figure S1). Conserved domain analysis results showed that all TaCCoAOMTs contained Caffeoyl coenzyme A 3-O-methyltransferase (CCoAOMT) AdoMet_MTases Superfamily domain (Figure 2B).

Gene family structure is a critical way to understand its evolution and functional diversity [23]. We analyzed the exon–intron structures of *TaCCoAOMT* genes using GSDS (Gene Structure Display Server). Most homologous *TaCCoAOMT* genes showed similar UTR and CDS distribution patterns. *TaCCoAOMT9*, *TaCCoAOMT17*, *TaCCoAOMT6* and *TaCCoAOMT1* were without UTR regions, and *TaCCoAOMT8* only contained the 3′ UTR region. In addition, *TaCCoAOMT5* and *TaCCoAOMT17* lacked intron regions (Figure 2C).

### 2.4. Chromosomal Distribution of TaCCoAOMT Genes

**Figure 3:**
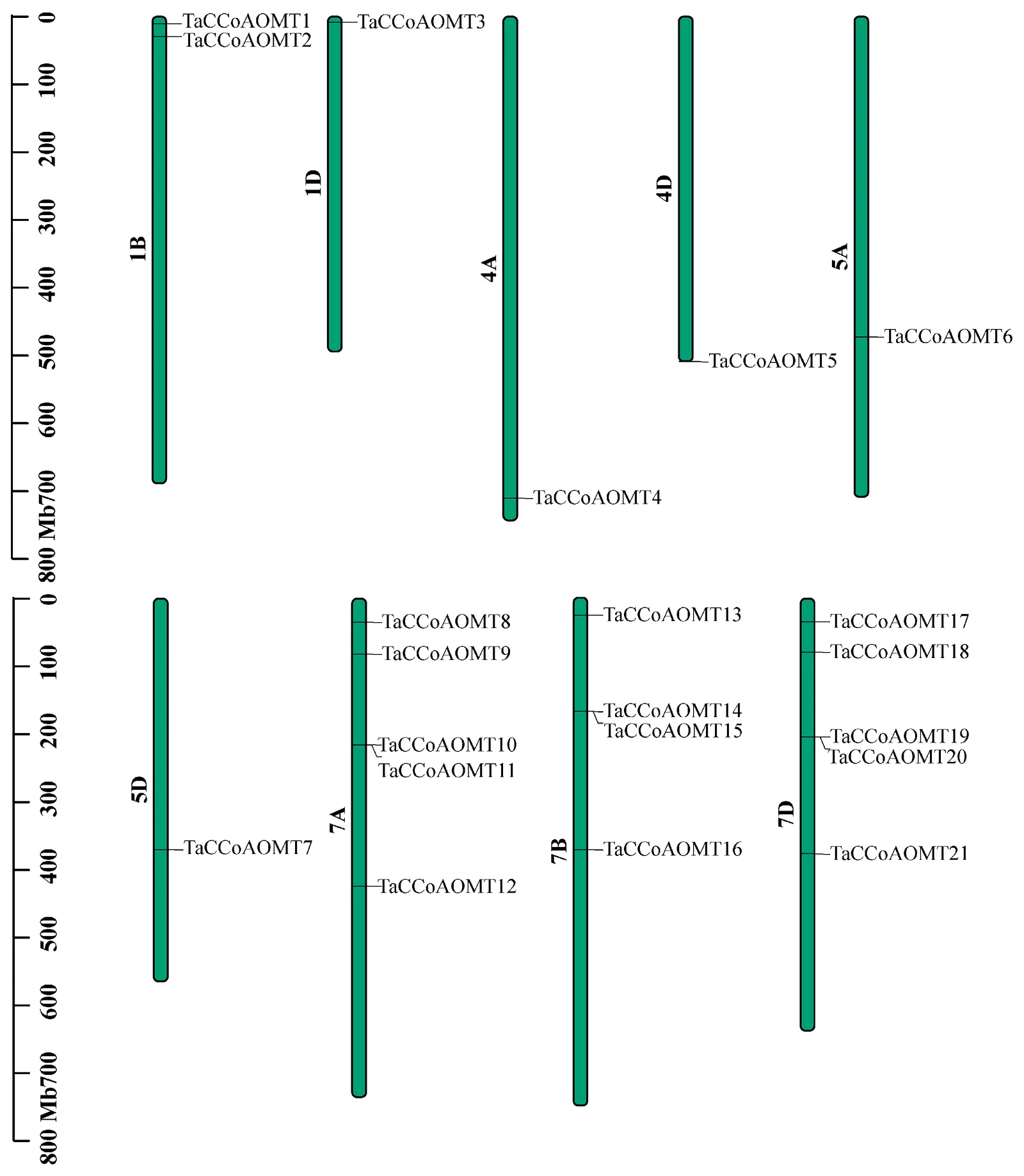
Chromosome localization of *TaCCoAOMT* genes in wheat.

Chromosomal mapping revealed that the 21 *TaCCoAOMT* genes were unevenly dispersed across nine wheat chromosomes. Chromosomes 7A, 7B, and 7D contained most of the *TaCCoAOMT* members, whereas the other chromosomes each carried only one or two genes (Figure 3).

### 2.5. Synteny Analysis of TaCCoAOMT Genes

**Figure 4:**
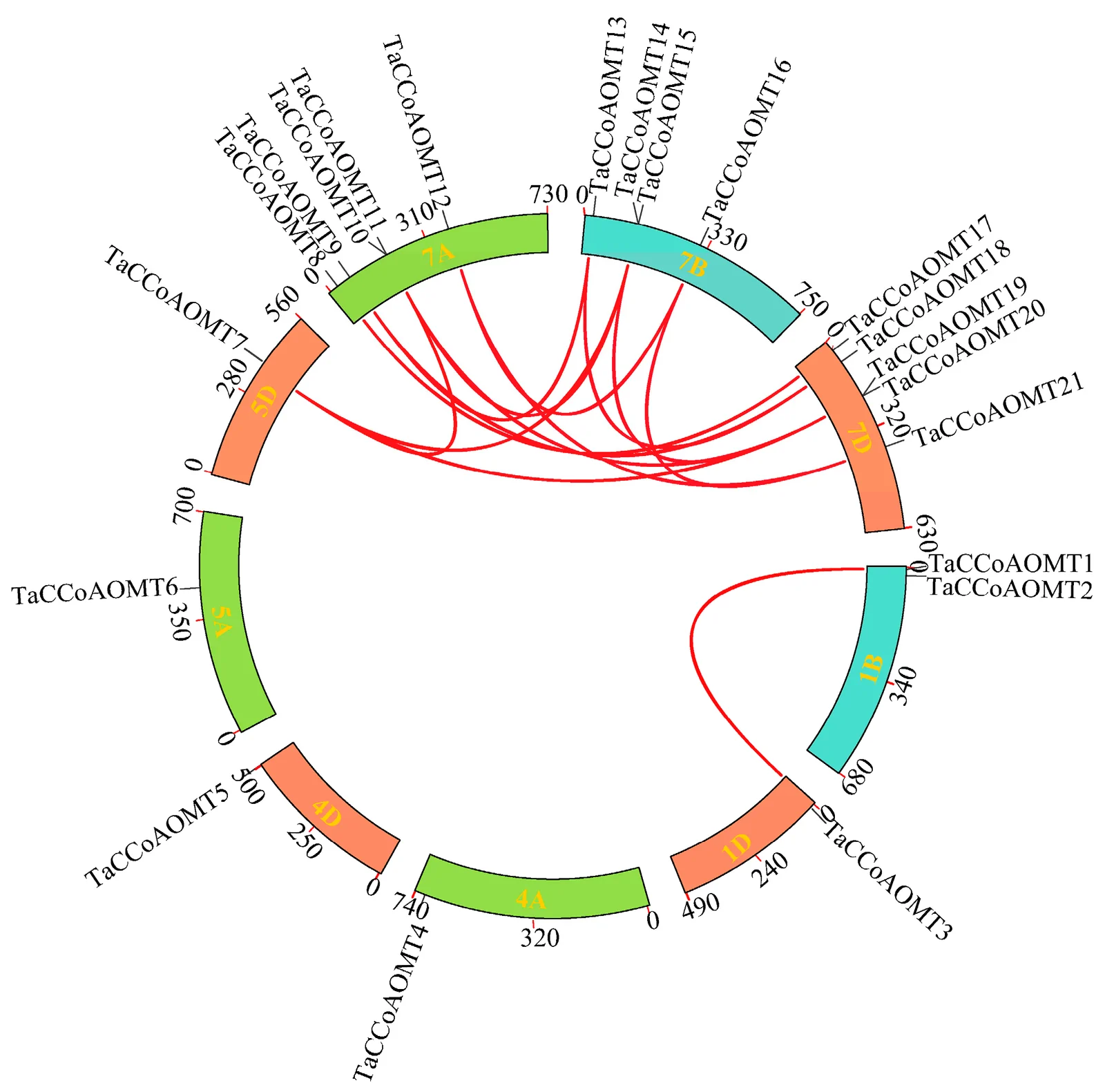
Collinearity analysis of *TaCCoAOMT* genes. The red lines represent the gene pairs.

Duplicate events contribute to gene family expansion and the acquisition of new functions. We performed collinearity analysis within the *TaCCoAOMT* family. Based on synteny analysis results, we identified thirteen gene pairs in the *TaCCoAOMT* family (Figure 4). The thirteen pairs in Table 1 are segmental duplications. Twelve of them are A/B/D homoeologous pairs. In addition, three tandem duplication pairs (*TaCCoAOMT10/11*, *TaCCoAOMT14/15*, and *TaCCoAOMT19/20*) were identified based on their adjacent locations on the same chromosomes. Both segmental and tandem duplication events contributed to the expansion of the *TaCCoAOMT* gene family. The Ka/Ks ratio directly indicates the selective pressures acting on a gene family over evolutionary time. Specifically, a Ka/Ks ratio greater than 1 signifies positive selection, a ratio less than 1 indicates negative (or purifying) selection, and a ratio equal to 1 reflects neutral selection [24]. The Ka, Ks and Ka/Ks of the *TaCCoAOMT* family gene pairs were calculated. All Ka/Ks values for these *TaCCoAOMTs* duplicated gene pairs were less than 1, indicating that the duplicated genes in the *TaCCoAOMT* family underwent purifying selection during evolution.

### 2.6. Analysis of Cis-Elements in TaCCoAOMT Gene Promoter Regions

The cis-acting element is a specific motif that regulates gene transcription and expression by interacting with different transcription factors [25]. We extracted 2000 bp promoter regions of *TaCCoAOMT* genes from the wheat genome and analyzed them using PlantCARE.

**Figure 5:**
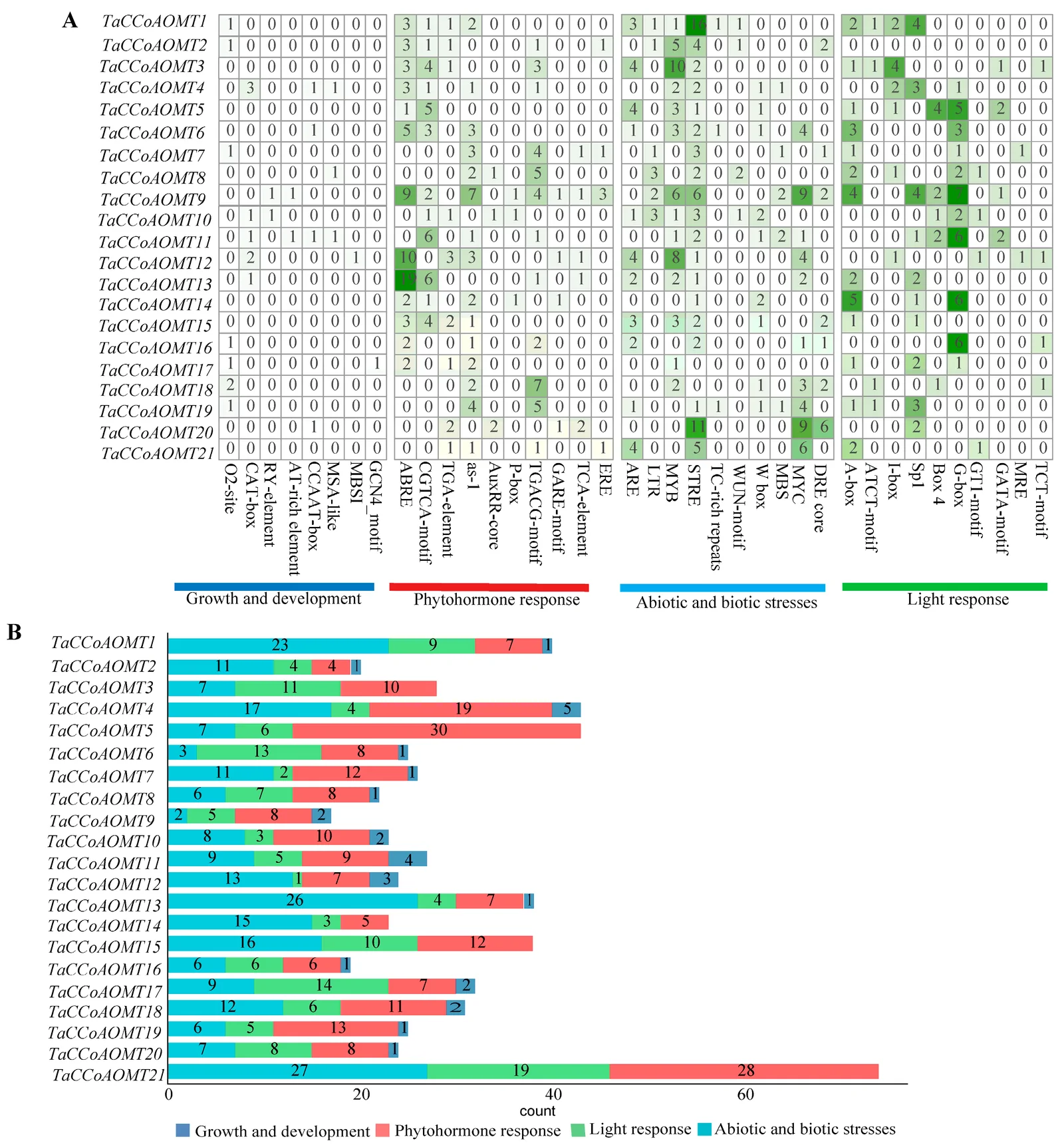
Prediction of cis-acting elements in *TaCCoAOMT* genes promoter regions. (**A**) The distribution of four category cis-acting elements in the *TaCCoAOMT* genes promoters. (**B**) The number of cis-acting elements of phytohormone response, growth and development, abiotic and biotic stress and light response in the *TaCCoAOMTs* promoters.

Promoter analysis predicted 1258 cis-acting elements in total (Figure 5). Among them, 667 were associated with growth and development, 145 with light responsiveness, 240 with biotic and abiotic stress responses, and 206 with phytohormone signaling. CAAT-box, TATA-box, and O_2_-site (a motif associated with zein metabolism regulation) were the most abundant cis-elements in the growth and development category. Abiotic and biotic-related cis-acting elements include anaerobic induction (ARE), low-temperature responsiveness (LTR), STRE and MYB. Cis-acting elements involved in phytohormone response include abscisic acid responsiveness (ABRE), gibberellin-responsive element (GARE-motif and P-box), and MeJA-responsiveness (CGTCA-motif and TGACG-motif). These results suggest that *TaCCoAOMT* genes may be associated with wheat growth and development.

### 2.7. Expression Profile of TaCCoAOMT Genes in Wheat

**Figure 6:**
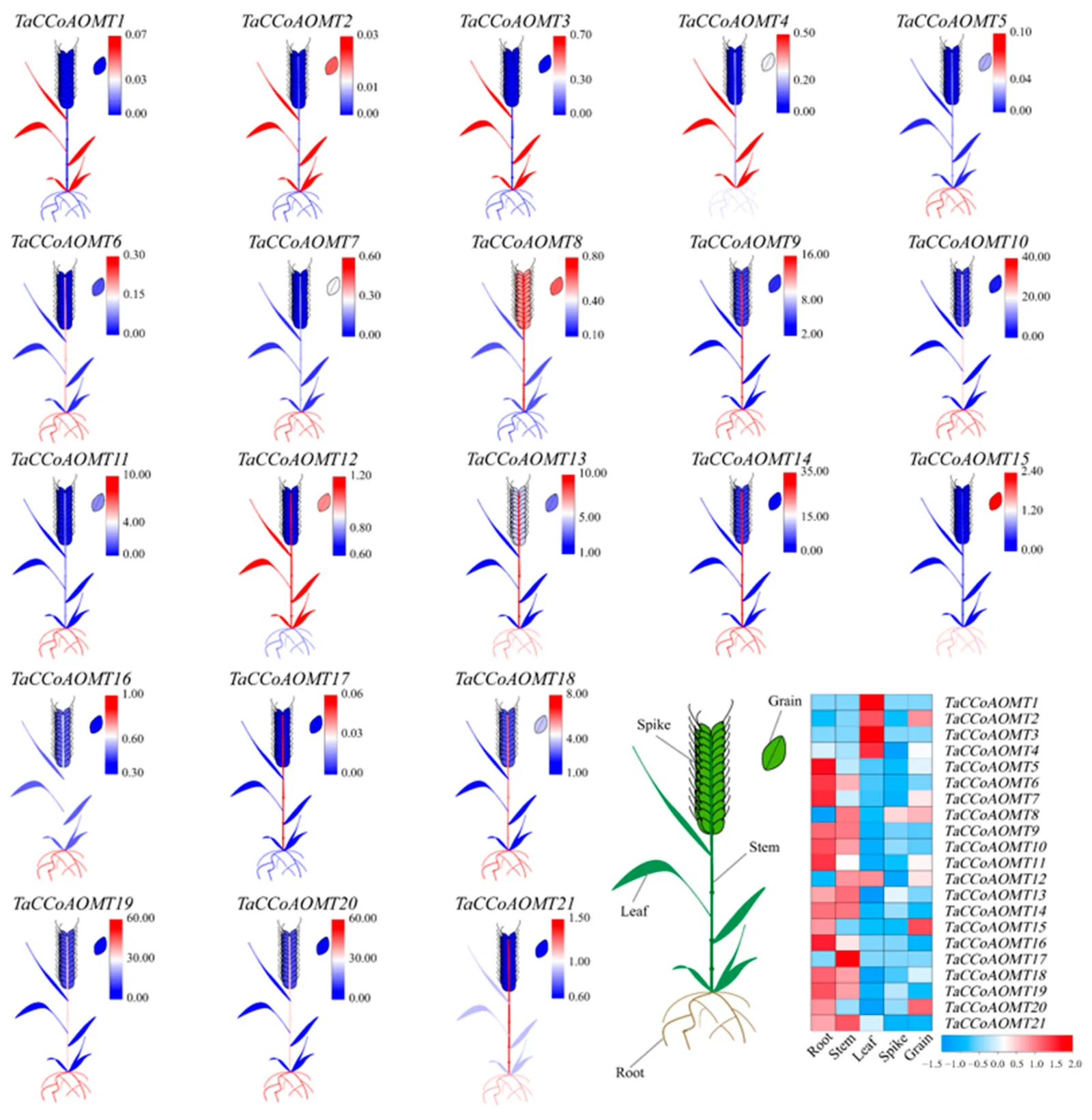
Transcription level of *TaCCoAOMT* genes in different tissues. The different colors of the heatmap indicate the level of transcription.

Expression pattern analysis helps explore the biological function of genes. In this study, we downloaded the RNA-Seq data from the Wheat URGI database. As shown in Figure 6, the *TaCCoAOMT* genes have tissue specificity. Compared with other members, *TaCCoAOMT9*, *TaCCoAOMT10*, *TaCCoAOMT11*, *TaCCoAOMT13*, *TaCCoAOMT14*, *TaCCoAOMT18*, and *TaCCoAOMT19* showed high expression levels. *TaCCoAOMT9*, *TaCCoAOMT10*, *TaCCoAOMT11*, *TaCCoAOMT13*, *TaCCoAOMT14*, *TaCCoAOMT18*, and *TaCCoAOMT19* were highly expressed in lignified tissues, specifically in the roots and stems (Figure 6). This result suggested that *TaCCoAOMT* genes may be involved in diverse biological processes.

### 2.8. Expression Levels Analysis of TaCCoAOMT Genes in Different Lodging-Resistant Wheat Cultivars

**Figure 7:**
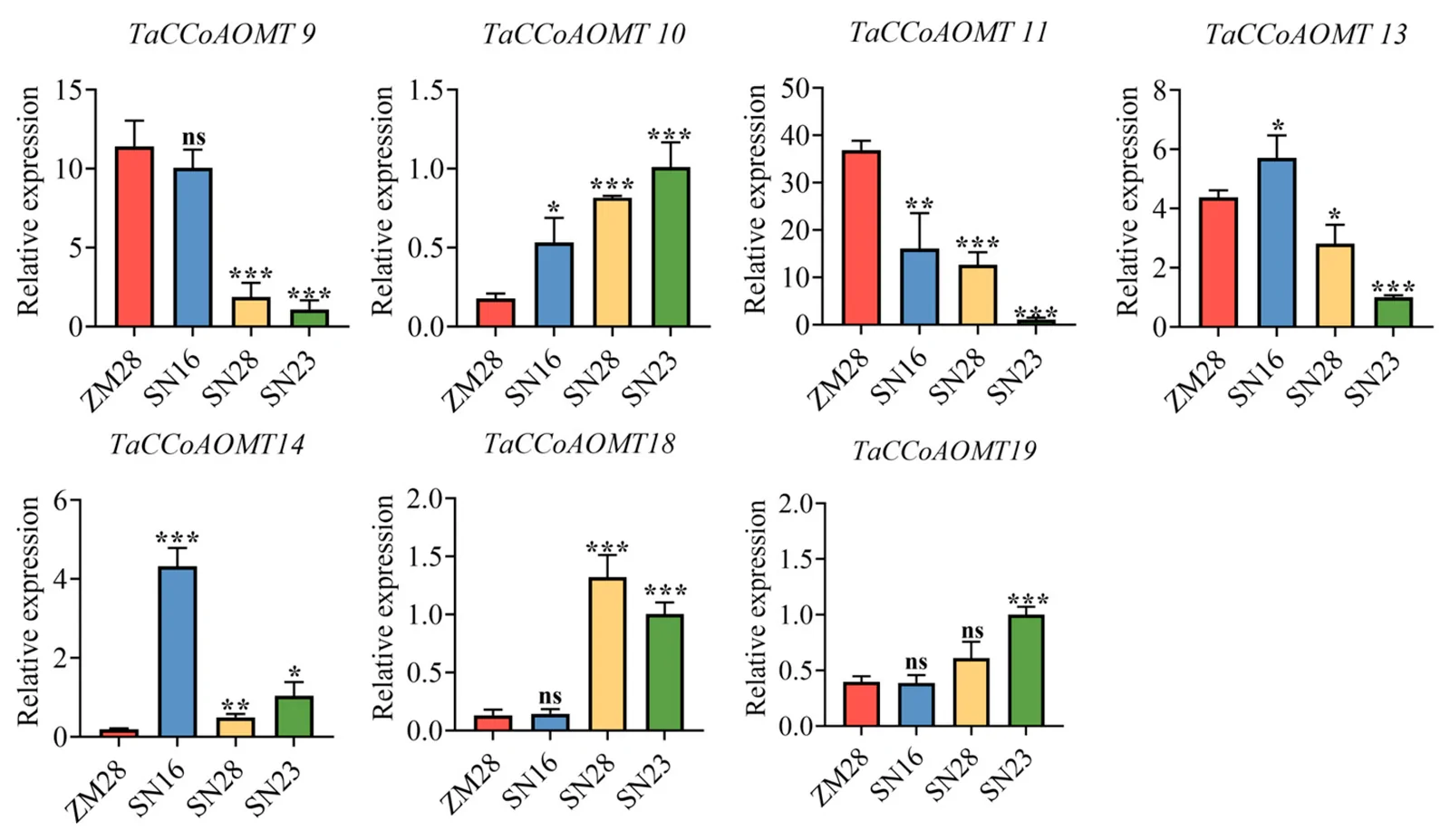
Relative expression levels of *TaCCoAOMT* genes among wheat cultivars with different lodging tolerance. SN23 was used as the calibrator for all genes, and its relative expression value was set to 1 for each gene. The y-axis scales differ among genes because of differences in absolute expression levels; all values are relative to the same calibrator (SN23). Vertical bars above mean values indicate standard deviations (*n* = 3 biological replicates). Means show significant differences between treatments according to the LSD (*, *p* < 0.05; **, *p* < 0.01; ***, *p* < 0.001; ns, not significant).

To examine the role of *TaCCoAOMT* genes in wheat stem lodging resistance, we carried out qRT-PCR analysis of *TaCCoAOMT* genes (*TaCCoAOMT9*, *TaCCoAOMT10*, *TaCCoAOMT11*, *TaCCoAOMT13*, *TaCCoAOMT14*, *TaCCoAOMT18*, and *TaCCoAOMT19*) in the lodging-resistant and sensitive wheat cultivars. *TaCCoAOMT10* and *TaCCoAOMT18*, exhibited high expression levels in the lodging-resistant varieties (SN28 and SN23) (Figure 7). According to the above findings, the expression levels of *TaCCoAOMT10* and *TaCCoAOMT18* may be positively correlated with stem lodging resistance; however, this association does not establish causality, and functional validation is required.

### 2.9. Differences in Stem Breaking Strength, Lignin and Lignin Monomer Content Among Different Varieties

**Figure 8:**
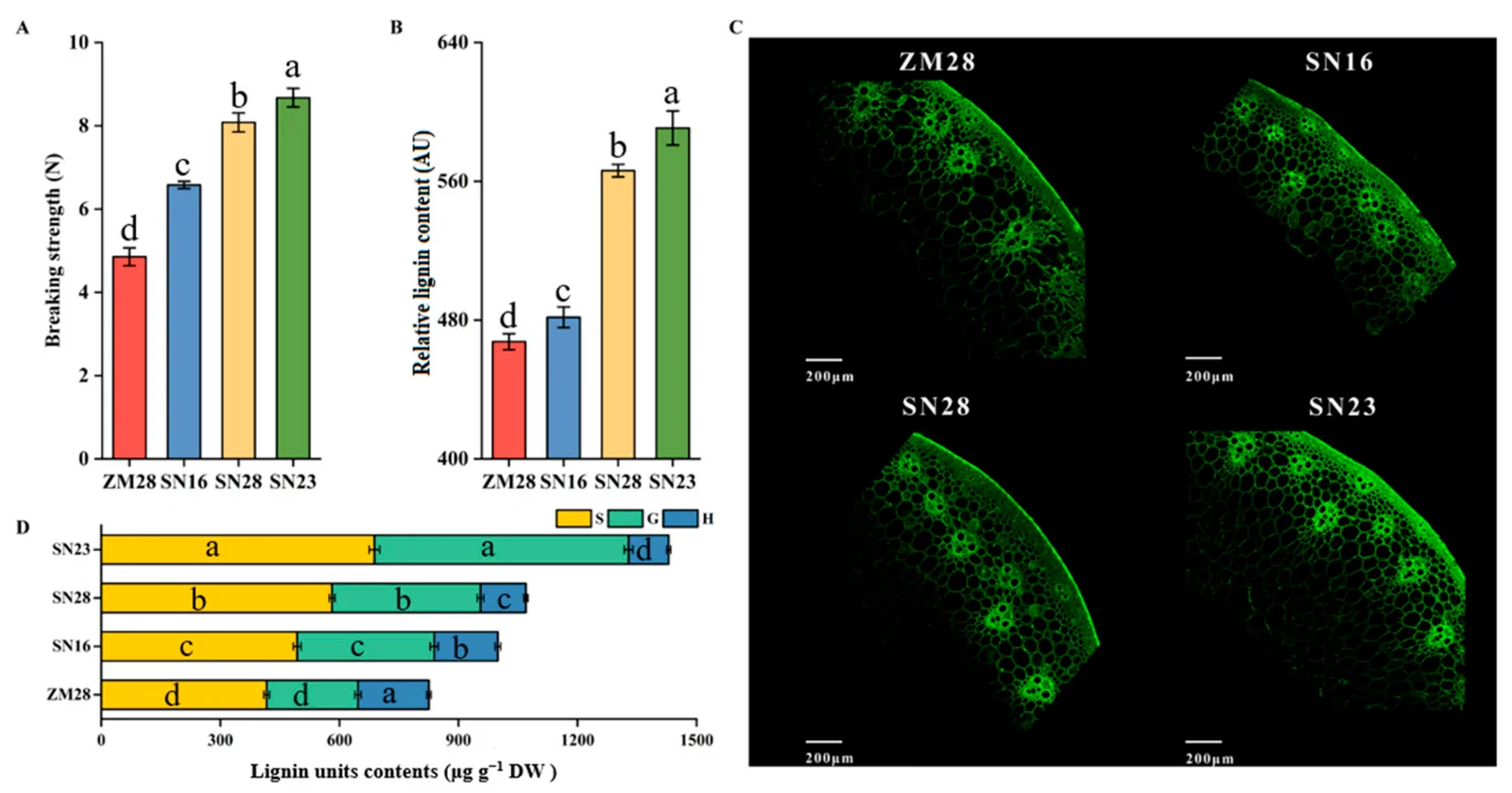
The stem traits of different lodging-tolerant wheat varieties. (**A**) Breaking strength of wheat second internodes (*n* = 10). Different lowercase letters above the bars indicate significant differences among cultivars (*p* < 0.05, LSD multiple-range test). (**B**) Relative lignin content in the second internode determined by the acetyl-bromide assay (*n* = 3). Values are expressed as arbitrary units (AU) for relative comparison among cultivars and do not represent the absolute mass percentage of lignin in dry weight. Different lowercase letters above bars indicate significant differences among cultivars (*p* < 0.05, LSD multiple-range test). (**C**) Lignin autofluorescence of second-internode cross-sections from ZM28, SN16, SN28, and SN23. Scale bar = 200 μm. (**D**) S, G, and H monomer contents of wheat stems (*n* = 3). Error bars indicate standard deviations. Different lowercase letters next to the bars indicate significant differences among cultivars (*p* < 0.05, LSD multiple-range test).

Stem breaking strength, lignin accumulation and lignin monomers are essential parameters for measuring stem lodging resistance. At the basal second internodes, SN23 exhibited the highest breaking strength, followed by SN28, SN16, and ZM28 in descending order. SN23 was significantly higher than SN28 (*p* < 0.05), SN28 was significantly higher than SN16 (*p* < 0.05), and SN16 was significantly higher than ZM28 (*p* < 0.05) (Figure 8A). Meanwhile, relative lignin content differed significantly among the four varieties: SN23 > SN28 > SN16 > ZM28 (Figure 8B). On the other hand, the outcome was further validated by lignin autofluorescence. Cultivars with enhanced resistance to lodging exhibited heightened lignin autofluorescence (Figure 8C). Moreover, the composition of lignin monomers also exhibits differences. Compared to SN16 and ZM28, SN23 and SN28 have higher S and G but lower H (Figure 8D).

### 2.10. Correlation Analysis of Breaking Strength, Lignin, and TaCCoAOMT Gene Expression

**Figure 9:**
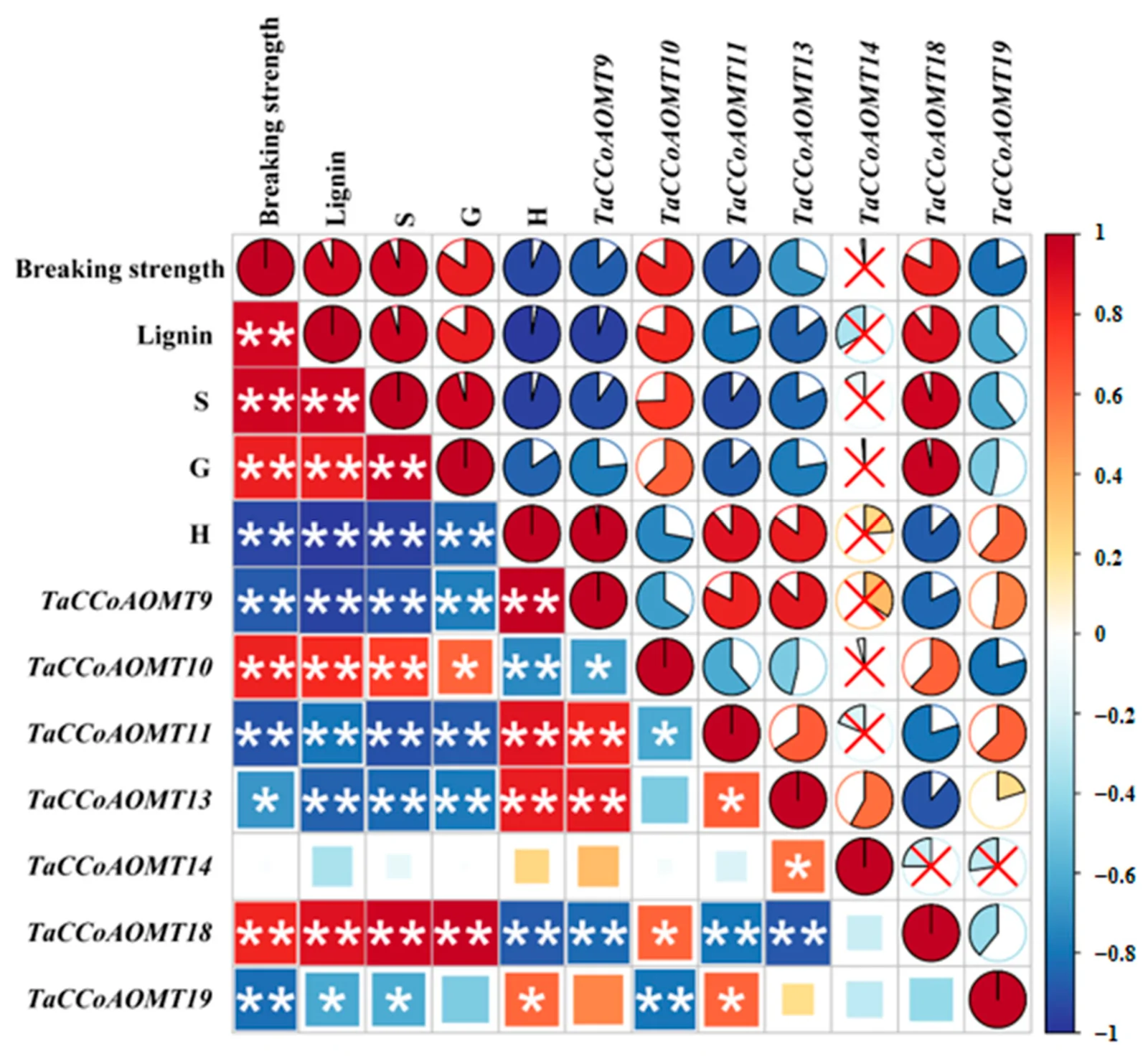
Pearson correlation matrix showing suggestive associations between lodging-related stem traits and *TaCCoAOMT* relative gene expression. Correlations were calculated using three matched biological replicates per cultivar for four cultivars (*n* = 12, *df* = 10). Note that replicates originating from the same cultivar are not statistically independent; correlations represent associative trends and cannot prove functional causality. * and ** indicate significance at *p* < 0.05 and *p* < 0.01, respectively. Red X-shaped symbols represent not significant at *p* < 0.05. The color gradient represents the Pearson correlation coefficient. Red segments indicate positive correlation coefficients, and blue segments indicate negative correlation coefficients. The stronger the correlation, the larger the rectangle and sector areas. Exact *r* and *p* values for the main gene–trait pairs are as follows: *TaCCoAOMT10* vs. breaking strength (*r* = 0.72, *p* = 0.008), *TaCCoAOMT10* vs. S monomer (*r* = 0.70, *p* = 0.011), *TaCCoAOMT10* vs. G monomer (*r* = 0.62, *p* = 0.033), *TaCCoAOMT10* vs. H monomer (*r* = −0.77, *p* = 0.003), *TaCCoAOMT18* vs. breaking strength (*r* = 0.68, *p* = 0.015), *TaCCoAOMT18* vs. S monomer (*r* = 0.58, *p* = 0.047), *TaCCoAOMT18* vs. G monomer (*r* = 0.60, *p* = 0.039), *TaCCoAOMT18* vs. H monomer (*r* = −0.71, *p* = 0.009), and *TaCCoAOMT19* vs. G monomer (*r* = 0.31, *p* = 0.326).

Correlation analysis in Figure 9 showed that *TaCCoAOMT10* and *TaCCoAOMT18* were positively correlated with breaking strength, as well as S and G monomers, and negatively correlated with H monomers (*p* < 0.05). *TaCCoAOMT14* did not differ significantly in breaking strength or lignin content (*p* > 0.05), while *TaCCoAOMT19* showed no significant association with G monomer levels (*p* > 0.05). The remaining *TaCCoAOMT9*, *TaCCoAOMT11*, and *TaCCoAOMT13* were negatively correlated (*p* < 0.05) with breaking strength or lignin content. These results suggest that *TaCCoAOMT10* and *TaCCoAOMT18* are associated with stem quality through lignin monomer biosynthesis.

### 2.11. Subcellular Localization Analysis

**Figure 10:**
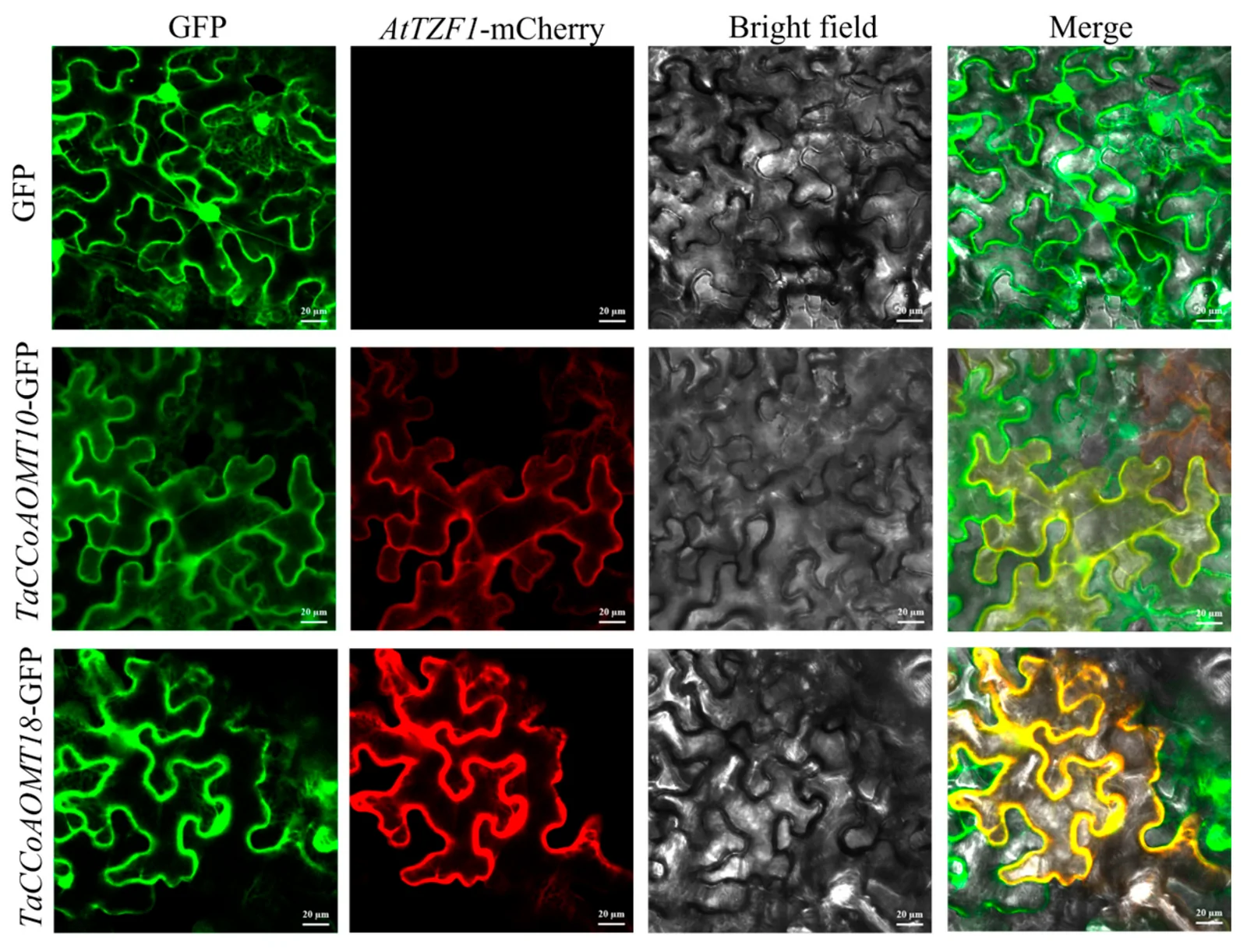
Subcellular localization of *TaCCoAOMT10* and *TaCCoAOMT18* in *Nicotiana benthamiana*. AtTZF1 was used as a cytoplasm marker. Bars= 20 μm.

Based on the correlation analysis results, we further investigated subcellular localization. *TaCCoAOMT10* and *TaCCoAOMT18* were introduced into the pBIN-GFP vector under the regulation of the CaMV35S promoter. The plasmid was introduced into *Nicotiana benthamiana* leaf epidermal cells using the *Agrobacterium tumefaciens*-mediated transformation method. After 48 h of incubation, free GFP was dispersed throughout the cells. In contrast, the TaCCoAOMT10–GFP and TaCCoAOMT18–GFP fusion proteins were predominantly localized in the cytoplasm and exhibited colocalization with AtTZF1–mCherry (Figure 10).

## 3. Discussion

CCoAOMT was discovered in 1989 from suspension cell cultures of parsley (*Petroselinum crispum*) and was subsequently shown to be a critical enzyme for lignin biosynthesis in zinnia (*Zinnia elegans*) and *Nicotiana benthamiana* [26,27,28]. Lignin deposited in the secondary cell wall provides plants with structural support, rigidity, and compressive strength [29,30]. CCoAOMT is involved in the phenylpropanoid pathway of lignin metabolism. After that, CCoAOMT has been isolated from various plants, such as arabidopsis (*Arabidopsis thaliana*) [31], rice (*Oryza sativa*) [32], sorghum (*Sorghum bicolor*) [33], jute (*Corchorus*) [34], and switchgrass (*Panicum virgatum*) [35]. Here, we identified 21 *TaCCoAOMT* genes in the wheat genome. These *TaCCoAOMTs* cluster into four subfamilies, similar to tea and jute *CCoAOMTs* (Figure 1) [36,37]. TaCCoAOMT proteins differ in physical and chemical properties, isoelectric point, molecular weight, and grand average hydropathy, indicating high genetic diversity in TaCCoAOMTs (Table S2).

The conservation and diversity of gene structures contribute to the evolution of genetic redundancy and novel functions. Within the same subgroup, most *TaCCoAOMT* genes share similar gene structures and conserved motifs (Figure 2). Conserved gene structures and motifs may be essential for their specific biological functions. Variations in gene structure and conserved motifs among different subgroups provide the basis for the emergence of new functions. Gene family expansion can arise from several mechanisms, including whole-genome duplication, tandem duplication, and segmental duplication [38]. This study found that *TaCCoAOMT* genes were unevenly distributed across the nine chromosomes (Figure 3). To test whether this uneven distribution reflected true homoeolog loss rather than an annotation artifact, we performed a sensitivity re-analysis using relaxed HMMER and BLASTP thresholds and checked for gaps in the IWGSC RefSeq v2.2 assembly. The results supported genuine homoeolog loss rather than an annotation artifact. This indicates that the asymmetric distribution of *TaCCoAOMT* genes among homoeologous chromosomes is likely due to homoeolog loss during wheat evolution.

Cis-acting elements directly or indirectly regulate gene expression, which is critical for gene biological function [39]. *OsCCoAOMT1* and *OsCCoAOMT2* mediate lignin synthesis, which is critical for rice adaptation to copper excess [19]. In the promoter region of the *TaCCoAOMT* genes, numerous MYB motifs have been identified (Figure 5A), which are closely associated with lignin biosynthesis [40]. Previous reports have demonstrated that various phytohormones regulate lignin biosynthesis. In rice, Gibberellin (GA) enhances the expression of the lignin biosynthesis gene 4-COUMARATE: COENZYME A LIGASE 3 (4CL3) by modulating SLENDER RICE1 (SLR1), thereby promoting lignin accumulation [41]. Promoter cis-element analysis indicated that *TaCCoAOMT* genes may participate in multiple biological pathways in wheat, including growth and development, phytohormone signaling, and stress responses (Figure 5). This finding corroborates earlier investigations.

To further analyze the function of *TaCCoAOMT* genes, we examined their expression in different tissues. Most *TaCCoAOMT* members were highly expressed in the stem or root with high lignification (Figure 6). A similar result of the expression pattern has already been reported in Sorghum [33]. This result suggests that most *TaCCoAOMT* genes are related to stem or root development. After that, we identified seven *TaCCoAOMT* genes that exhibited high expression levels in the stem (Figure 7). Previous reports indicate that lignin synthesis occurs within the cytoplasm [42,43]. Subcellular localization showed that *TaCCoAOMT10* and *TaCCoAOMT18* were located in the cytoplasm (Figure 10), which aligns with prior research [44,45].

CCoAOMT catalyzes the methylation of caffeoyl-CoA and 5-hydroxyferuloyl-CoA to produce feruloyl-CoA and sinapoyl-CoA, which are precursors of G and S monolignols [16,17,18]. In this study, *TaCCoAOMT10* and *TaCCoAOMT18* showed higher expression in lodging-resistant cultivars and were positively correlated with breaking strength, lignin content, and S/G monomer contents, while being negatively correlated with H monomer content (Figure 7, Figure 8 and Figure 9). Their cytoplasmic localization is consistent with the subcellular site of monolignol biosynthesis [42,43]. These associations suggest that *TaCCoAOMT10* and *TaCCoAOMT18* may contribute to lignin deposition and stem mechanical strength by promoting S/G monolignol biosynthesis. Consistent with these molecular patterns, lodging-resistant cultivars had higher breaking strength and lignin content and higher S/G but lower H monomer proportions than lodging-sensitive cultivars (Figure 8). However, correlation alone does not establish causality; overexpression, knockout, or VIGS experiments are required to determine whether these genes causally regulate lodging-related traits. The four cultivars used here represent lodging-resistant and lodging-sensitive genotypes. Plant height, stem diameter, and other morphological traits may also contribute to the observed differences in breaking strength and lignin content; therefore, the roles of lignin and *CCoAOMT* are interpreted as associations rather than sole causes. Future studies can further quantify these traits to provide additional morphological context. Taken together, this study provides associative evidence that *TaCCoAOMT* gene family members are associated with lignin biosynthesis and wheat lodging resistance. *TaCCoAOMT10* and *TaCCoAOMT18* are identified as candidate genes, but causal roles cannot be established from correlation alone. Functional validation by overexpression, knockout, or VIGS is required in future studies.

## 4. Materials and Methods

### 4.1. Identification and Sequence Analysis of Wheat CCoAOMT Family Members

The whole-genome, protein sequence, and gene annotation files of wheat (IWGSC RefSeq v2.2), rice and Arabidopsis were obtained from Ensembl Plants (http://plants.ensembl.org/index.html, accessed on 12 August 2024) [46]. The Hidden Markov Model (HMM) file corresponding to the SAM-dependent O-methyltransferase structure domain (PF01596) was acquired from the website of PFAM (https://www.ebi.ac.uk/Tools/hmmer/search/phmmer, accessed on 12 August 2024) [47]. Using the HMM profile and conserved domain analysis, we identified *TaCCoAOMT* family members from the wheat genome dataset, with the HMMER search performed at an *E*-value threshold of 1 × 10^−5^. Chromosomal locations of *TaCCoAOMT* genes were retrieved from the IWGSC RefSeq v2.2 annotation [48], and protein molecular weights were calculated from the protein sequences. The physicochemical parameters of TaCCoAOMT proteins, including their isoelectric points (pI), instability indices (II), and grand average of hydropathicity (GRAVY) values, were computed using the ProtParam tool hosted on the ExPASy server (https://web.expasy.org/protparam/, accessed on 12 August 2024) [49]. All gene IDs in Table S2 are based on the IWGSC RefSeq v2.2 annotation.

### 4.2. Phylogenetic Analysis of TaCCoAOMTs

Multiple sequence alignments of the TaCCoAOMT, OsCCoAOMT and AtCCoAOMT proteins were accomplished through MEGA 11.0 [50]. Next, MEGA built a phylogenetic tree using the Maximum likelihood method (ML). The phylogenetic tree was beautified by the online program iTOL (http://itol.embl.de) [51].

### 4.3. Gene Structure, Conserved Domain, and Conserved Motifs Analysis

Conserved motifs of TaCCoAOMTs were detected using the MEME Suite web server (version 5.5.5; https://meme-suite.org/meme/tools/meme, accessed on 12 August 2024), with the number of motifs to be identified limited to fifteen [52]. Conserved domain analysis of TaCCoAOMT proteins using NCBI Batch CD-Search. For visualization, the gene structure and motif organization data were processed through the Visualize Gene Structure module of TBtools (version v1.0987) [53].

### 4.4. Chromosomal Mapping of TaCCoAOMT Genes in Wheat Genome

The Gene Location Visualization tool of TBtools was employed to map and display the chromosomal distribution of *TaCCoAOMT* genes according to the genome assembly.

### 4.5. Collinearity, Gene Duplication and Ka (Non-Synonymous Substitution)/Ks (Synonymous Substitution) Analysis

The collinearity analysis of *TaCCoAOMT* genes among wheat was performed with the One Step MCscanX plugin of TBtools software. The synteny results figure was generated by Advanced Circle in TBtools software. Gene duplications can be classified as tandem or segmental duplications. Segmental duplicated genes were defined based on the following criteria: (1) sequence alignment similarity > 80%, and (2) threshold ≤ 10^−10^ [54]. The ratio of Ka/Ks of paired genes was computed using the Simple Ka/Ks Calculator (NG) plugin. The magnitude of Ka/Ks reflects selection pressure during evolution [24].

### 4.6. Transcriptional Analysis of TaCCoAOMT Genes in Various Tissues

Transcriptomic data for *TaCCoAOMT* genes were obtained from the publicly accessible Wheat URGI 2.2 database (https://wheat-urgi.versailles.inra.fr/Projects/IWGSC, accessed on 12 August 2024) [55]. Transcriptome data were obtained from the transcriptional levels of five tissues of Chinese Spring wheat: root, stem, leaf, spike, and grain. The transcriptional levels were expressed in TPM. The heatmap was generated using TBtools (version v1.0987).

### 4.7. Plant Materials

Two lodging-resistant varieties, Shannon 23 (SN23) and Shannon 28 (SN28), and two lodging-sensitive varieties, Shannon 16 (SN16) and Zimai 28 (ZM28), were used in this study. These classifications are based on previous multi-year field studies [56,57], in which SN23 and SN28 were characterized as lodging-resistant and SN16 and ZM28 as lodging-sensitive. Previous studies have also characterized these cultivars for plant height, stem diameter, and other stem morphological traits.

The four cultivars were cultivated in the 2024–2025 growing season at Tai’an, Shandong Province, China (36°09′ N, 117°09′ E), in a randomized complete block design (RCBD) with three replicate plots per cultivar. Each plot was 3 m × 3 m. All samples for qRT-PCR, lignin measurement, lignin monomer quantification, and breaking strength determination were collected from these replicated plots. All phenotypic and molecular data in this study were obtained from this single 2024–2025 growing season. The experimental site has a warm-temperate continental semi-humid monsoon climate, with a multi-year average precipitation of approximately 698 mm (range 684.7–735.3 mm) and a multi-year average temperature of approximately 13.5 °C (range 13.0–14.2 °C).

Before planting, 120 kg ha^−1^ Nitrogen, 75 kg ha^−1^ P_2_O_5_, and 150 kg ha^−1^ K_2_O were applied, and 120 kg ha^−1^ nitrogen (urea 46%) was applied at the jointing stage (GS 31). The planting density was 225 plants m^−2^.

### 4.8. Total RNA Extraction and qRT-PCR Analysis

At anthesis (GS 65) [58], we collected the second basal internodes of four varieties and stored them at −80 °C. Next, the samples were ground in liquid nitrogen. Total RNA was isolated from plant samples with the FastPure Universal Plant Total RNA Isolation Kit (Vazyme, Nanjing, China) in accordance with the manufacturer’s instructions. First-strand cDNA was then generated from the purified RNA using the HiScript II 1st Strand cDNA Synthesis Kit (Vazyme, Nanjing, China). We performed qRT-PCR with the AceQ qPCR SYBR Green Master Mix (Vazyme, Nanjing, China). The primers used in this study are listed in Table S3. Primer specificity was verified by melt curve analysis, and each primer pair produced a single peak. The expression levels of target genes were normalized to a single *TaActin* reference gene. For each cultivar, three biological replicates were used, and each biological replicate was technically repeated three times. The relative gene expression level was calculated using the 2^−ΔΔCt^ method with SN23 as the calibrator for all genes. By definition, the relative expression value of each gene in SN23 was set to 1. For correlation analysis, the three biological replicates per cultivar were treated as individual data points, yielding 12 observations across the four cultivars (*n* = 12).

### 4.9. Determination of Breaking Strength

At the anthesis stage (GS 65), ten basal second internodes from each variety were collected. After removing the stem sheath, the stems were placed on the stem strength tester (YYD-1, Top Instrument, Hangzhou, China) [59].

### 4.10. Determination of Relative Lignin Content

Reference was made to Hussain et al. [60]. A 0.1 g dried stem sample was accurately weighed; 25% acetyl bromide (*v*/*v*) and perchloric acid were added, and the sample was bathed in 80 °C water. Then, 0.5 mL of the solution was transferred to a centrifuge tube, and 2 M NaOH and hydroxylamine hydrochloride were added. The absorbance at 280 nm was measured with a TU-1950 spectrophotometer (Purkinje General Instrument, Beijing, China) after adding glacial acetic acid to a 10 µL aliquot. Relative lignin content was estimated using the extinction coefficient for grass lignin at 280 nm (20.09 L g^−1^ cm^−1^) as: Relative lignin content = (A_280_ × V × dilution)/(ε × l × m) × 1000, where A_280_ is absorbance, V is the final volume (mL), dilution is the dilution factor, ε is the extinction coefficient (L g^−1^ cm^−1^), l is the path length (cm), and m is the sample weight (g). The values are reported in arbitrary units (AU) and are intended only for relative comparison among cultivars; they do not represent the absolute mass percentage of lignin in dry weight.

### 4.11. Determination of Lignin Monomer Contents

The content of lignin monomers (S, G, H) was determined by following Zheng et al. [61] with slight modifications. An ultra-performance liquid chromatography–electrospray tandem mass spectrometry (UPLC-MS/MS) system (ACQUITY UPLC I-Class/Xevo TQ-S, Waters, Milford, MA, USA) was used to qualitatively and quantitatively analyse the three lignin units from wheat stems. The dried samples of 20 mg of protein-free stems were accurately weighed in a 50 mL ablation tube; then 3 mL of 2 M NaOH and 0.5 mL of nitrobenzene were added, and then heated for 1 h at 170 °C in a Master 40 microwave ablator (Xinyi Microwave, Shanghai, China). The reaction mixture was then centrifuged, and the supernatant was extracted twice with 4 mL of ethyl acetate. The ethyl acetate was evaporated to dryness using a Concentrator plus vacuum centrifugal concentrator (Eppendorf, Hamburg, Germany). The sample was reconstituted with 6 mL of 50% acetonitrile (*v*/*v*), filtered through a 0.22 μm membrane, and injected in 4 μL for detection.

### 4.12. Observation of Lignin Autofluorescence

At the anthesis stage (GS 65), samples were selected from the middle 2 cm of the second internode at the base of the stem and fixed in fixative (38% form aldehyde: glacial acetic acid: 70% alcohol = 1:1:18; *v*/*v*/*v*). Cross-sections of the stems were cut using the freehand sectioning method and placed under a laser confocal microscope (TCS-SP8, Leica Microsystems, Wetzlar, Germany) to observe lignin autofluorescence [62]. Excitation light: 365 nm, filter 395 nm, and emission ≥ 420 nm. Samples were collected for lignin autofluorescence using the same laser intensity (5%) and gain value (650 a.u.).

### 4.13. Subcellular Localization Analysis

The full-length coding sequences (CDS) of *TaCCoAOMT10* and *TaCCoAOMT18* without the stop codon were fused into the pBIN-GFP vector, respectively. The full-length CDS of AtTZF1, which has proven to be located in the cytoplasm, was introduced into the pCAMBIA1300-mCherry vector [63,64]. The plasmids were introduced into *Nicotiana benthamiana* leaf epidermal cells using the *Agrobacterium tumefaciens*-mediated transformation method. After 48 h, fluorescence was monitored under an LSM880 confocal microscope (Carl Zeiss, Jena, Germany) [65]. The primers used in this experiment are listed in Table S3.

### 4.14. Statistical Analysis

Analyses were performed using IBM SPSS Statistics 22.0 (IBM Corp., Armonk, NY, USA). A one-way analysis of variance (ANOVA) followed by least-significant difference (LSD) multiple-comparison test was used for phenotypic and qRT-PCR data. Origin 2023 (OriginLab Corporation, Northampton, MA, USA) was used for graphing.

Pearson correlation analysis (Figure 9) was performed on three biological replicates per cultivar across four wheat cultivars (*n* = 12, *df* = 10). Breaking strength, lignin content, lignin monomer contents and gene expression data used for correlation were obtained from the same matched set of biological replicate plants. Additional plants were measured only for phenotypic ANOVA of breaking strength and were not included in correlation computation. Significance level was set at *p* < 0.05.

## 5. Conclusions

In summary, this study comprehensively evaluates the *CCoAOMT* gene family in the wheat genome. We identified 21 *TaCCoAOMT* members and classified them into four subgroups based on conserved gene structure and motifs. From an evolutionary perspective, we identified both segmental and tandem duplication events that contributed to the expansion of the *TaCCoAOMT* gene family. We further identified some *TaCCoAOMT* genes highly expressed in the root and stem. *TaCCoAOMT10* and *TaCCoAOMT18* were associated with wheat lodging-related traits and lignin monomer formation, and they are therefore considered candidate genes for further validation. However, the present evidence is associative only, and causal functions require overexpression, knockout, or VIGS validation. These findings provide useful information for the targeted modification of *TaCCoAOMT* genes using molecular techniques. This knowledge will support breeding new wheat varieties with improved lodging tolerance.

## Figures and Tables

**Table 1:** Ka, Ks and Ka/Ks analysis for duplicated *TaCCoAOMT* gene pairs.

Gene Name	Gene Name	Ka	Ks	Ka/Ks	Duplicate Type
*TaCCoAOMT1*	*TaCCoAOMT3*	0.04	0.17	0.23	Segmental
*TaCCoAOMT7*	*TaCCoAOMT10*	0.31	0.45	0.68	Segmental
*TaCCoAOMT7*	*TaCCoAOMT14*	0.33	0.46	0.70	Segmental
*TaCCoAOMT7*	*TaCCoAOMT19*	0.31	0.49	0.62	Segmental
*TaCCoAOMT9*	*TaCCoAOMT13*	0.01	0.04	0.16	Segmental
*TaCCoAOMT9*	*TaCCoAOMT18*	0.01	0.03	0.32	Segmental
*TaCCoAOMT10*	*TaCCoAOMT14*	0.01	0.04	0.19	Segmental
*TaCCoAOMT10*	*TaCCoAOMT19*	0.00	0.07	0.05	Segmental
*TaCCoAOMT12*	*TaCCoAOMT16*	0.01	0.02	0.80	Segmental
*TaCCoAOMT12*	*TaCCoAOMT21*	0.02	0.06	0.29	Segmental
*TaCCoAOMT13*	*TaCCoAOMT18*	0.01	0.05	0.17	Segmental
*TaCCoAOMT14*	*TaCCoAOMT19*	0.00	0.05	0.07	Segmental
*TaCCoAOMT16*	*TaCCoAOMT21*	0.02	0.05	0.38	Segmental

## Data Availability

The data presented in this study are available within the article and its Supplementary Information Files. The RNA-Seq data analyzed in this study are available from the Wheat URG1 2.2 database (https://wheat-urgi.versailles.inra.fr/Projects/IWGSC, accessed on 12 August 2024). Additional data are available from the corresponding author upon reasonable request.

## Supplementary Materials

The following supporting information can be downloaded at: https://www.mdpi.com/article/10.3390/plants15192996/s1, Figure S1: The amino acid sequences of conserved motifs. The integral height of the stacks indicates the degree of conservation at this site, and the size of letters represents the frequency of the corresponding amino acid. Table S1: The IDs and accession numbers of the TaCCoAOMT family genes in Arabidopsis and rice. Table S2: Characteristics of CCoAOMT genes in wheat. Table S3: Primers sequences in this study.
