# Supplementary material for: Genome-Wide Identification of the Caffeoyl Coenzyme A 3-O-Methyltransferase Family Genes and Their Role in the Lodging Resistance of Wheat (Triticum aestivum L.)
Source: Plants (Basel). 2026 Sep 30;15(19):2996. doi: 10.3390/plants15192996 (PMC13667043; doi:10.3390/plants15192996)
Supplement: Supplementary file 1 [file plants-15-02996-s001.zip › plants-4560534-supplementary.pdf]

Table S1 The IDs and accession numbers of the *TaCCoAOMT* family genes in Arabidopsis and rice.

ID	Accession number	ID	Accession number
AtCCoAOMT1	*AT4G34050.1*	*OsCCoAOMT1*	*Os06g0165800*
AtCCoAOMT2	*AT1G24735.1*	*OsCCoAOMT2*	*Os08g0498100*
AtCCoAOMT3	*AT1G67980.1*	*OsCCoAOMT3*	*Os08g0498400*
AtCCoAOMT4	*AT1G67990.1*	*OsCCoAOMT4*	*Os08g0154000*
AtCCoAOMT5	*AT3G61990.1*	*OsCCoAOMT5*	*Os08g0498600*
AtCCoAOMT6	*AT3G62000.1*	*OsCCoAOMT6*	*Os09g0481400-01*
AtCCoAOMT7	*AT4G26220.1*		

Table S2 Characteristics of *CCoAOMT* genes in wheat.

Name	ID	Chromosome Location	AA	Mw (kDa)	pI	Ins	GRAVY
TaCCoAOMT1	*TraesCS1B02G023200.1*	1B:10414427-10416514	186	20.34	10.22	63.87	-0.732
TaCCoAOMT2	*TraesCS1B02G049800.1*	1B:29491770-29492937	210	23.35	5.55	25.39	-0.063
TaCCoAOMT3	*TraesCS1D02G019000.1*	1D:8259962-8261206	248	27.03	5.02	27.85	0.139
TaCCoAOMT4	*TraesCS4A02G442400.1*	4A:710393534-710394888	246	27.05	4.98	32.16	-0.121
TaCCoAOMT5	*TraesCS4D02G362500.1*	4D:508806906-508807847	190	20.39	5.04	42.44	0.176
TaCCoAOMT6	*TraesCS5A02G257600.1*	5A:472589923-472591058	252	27.30	5.39	34.88	0.142
TaCCoAOMT7	*TraesCS5D02G265900.1*	5D:370305798-370307383	242	26.19	5.15	35.77	0.136
TaCCoAOMT8	*TraesCS7A02G068600.1*	7A:34721320-34722967	288	31.89	7.01	40.77	-0.252
TaCCoAOMT9	*TraesCS7A02G127600.1*	7A:81858717-81859809	284	31.49	5.04	34.17	-0.249
TaCCoAOMT10	*TraesCS7A02G240300.1*	7A:215545815-215547567	247	27.09	4.99	29.82	-0.084
TaCCoAOMT11	*TraesCS7A02G240400.1*	7A:215879353-215880571	245	27.07	4.95	35.18	-0.145
TaCCoAOMT12	*TraesCS7A02G302500.1*	7A:424315801-424320367	300	32.89	8.59	54.26	-0.100
TaCCoAOMT13	*TraesCS7B02G027200.1*	7B:26024015-26025743	267	29.51	5.11	29.26	-0.197
TaCCoAOMT14	*TraesCS7B02G135900.1*	7B:167574529-167576382	302	33.14	5.54	30.27	-0.115
TaCCoAOMT15	*TraesCS7B02G136000.1*	7B:167686223-167687956	245	27.12	4.89	33.43	-0.190
TaCCoAOMT16	*TraesCS7B02G202600.1*	7B:371733486-371737703	294	32.15	7.57	56.91	-0.071
TaCCoAOMT17	*TraesCS7D02G063100.1*	7D:34337259-34337705	148	16.28	11.11	85.66	-0.866
TaCCoAOMT18	*TraesCS7D02G126000.1*	7D:78774437-78776901	260	28.86	5.11	31.31	-0.201
TaCCoAOMT19	*TraesCS7D02G239200.1*	7D:203947110-203948657	247	27.07	4.98	29.48	-0.081
TaCCoAOMT20	*TraesCS7D02G239400.1*	7D:204086773-204087957	245	27.12	4.94	31.84	-0.161
TaCCoAOMT21	*TraesCS7D02G297800.1*	7D:376559830-376563657	311	34.16	8.38	59.58	-0.124

AA, amino acids; MW, molecular weight; pI, isoelectric point; Ins, instability index; GRAVY, Grand average of hydropathicity. All gene IDs are based on the IWGSC RefSeq v2.2 annotation.

Table S3 Primers sequences in this study

Primer name	Forward primers (5' to 3')	Reverse primers (5' to 3')	Usage
Actin	AGGTGCCCTGAGGTGCTGTT	GCCAAAATAGAGCCACCGAT	qRT-PCR
TaCCoAOMT9	CACCGTAAGCTCCCTCACT	AGCTCGTAGTTCTCGCGGTT	qRT-PCR
TaCCoAOMT10	ACCACCGTCTTCCACCGT	GTCCGACGACGACTTCATGT	qRT-PCR
TaCCoAOMT11	GACGACCTCTACGACTACATGC	CGACAGCAGCAGCCTCTCA	qRT-PCR
TaCCoAOMT13	GTTCCCAAGGAGCAGCA	CCAGGATGTACTGGTAGA	qRT-PCR
TaCCoAOMT14	TACAAGGCGCTGGTTGGT	GGTGGTAGGAAGGAATGA	qRT-PCR
TaCCoAOMT18	ACCACCACCTGTTGACTG	GATTGGATTGACGAGCTT	qRT-PCR
TaCCoAOMT19	CAGAACTAGCGAGCAGAG	GAGGATGTAAGTGTAGAG	qRT-PCR
TaCCoAOMT10-SUB	ttacgaacgatagccggtaccATGGCGCCCAACGGAGACAA	gctcaccatcccgggggtaccGACGAGGCGGCGGCAGAT	Construction of *TaCCoAOMT10* subcelluar vector
TaCCoAOMT18-SUB	ttacgaacgatagccggtaccATGGCGACCACGGCAGCC	gctcaccatcccgggggtaccGACGAGGCGGCGGCAGAT	Construction of *TaCCoAOMT18* subcelluar vector
AtTZF1-mChe	agctcggtacccggggatccATGATGATCGGCGAAAATAAAAA	gaaaccattccccctctagaACCGAGTGAGTTCTCTCTAC	Construction of *AtTZF1* subcelluar vector

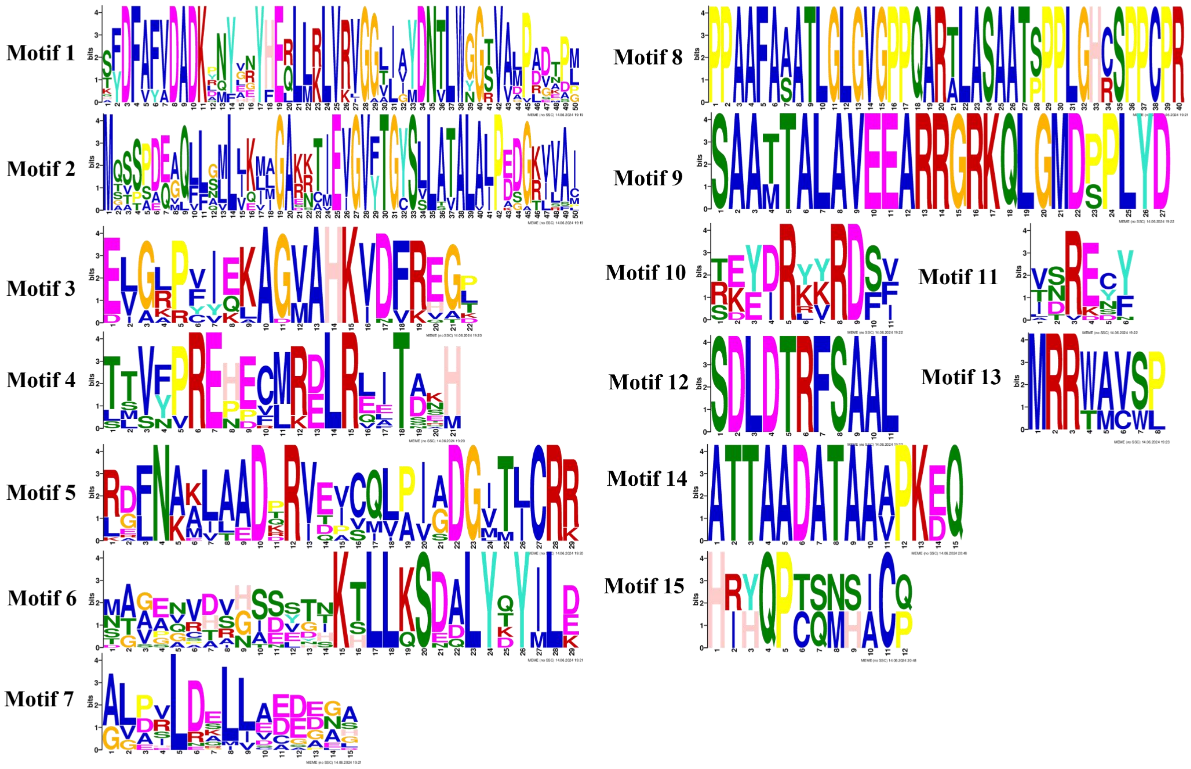

Figure S1. The amino acid sequences of conserved motifs. The integral height of the stacks indicates the degree of conservation at this site, and the size of letters represents the frequency of the corresponding amino acid.
